# Supplementary material for: Natural killer cell–mediated cytotoxicity shapes the clonal evolution of B cell leukaemia
Source: Cancer Immunol Res. Author manuscript; Available in PMC 2025 Jan 14. (PMC7617306; doi:10.1158/2326-6066.CIR-24-0189)
Supplement: Supplementary Materials [file EMS201860-supplement-Supplementary_Materials.zip › supp_info_12.docx]

# Supplementary Figure S10


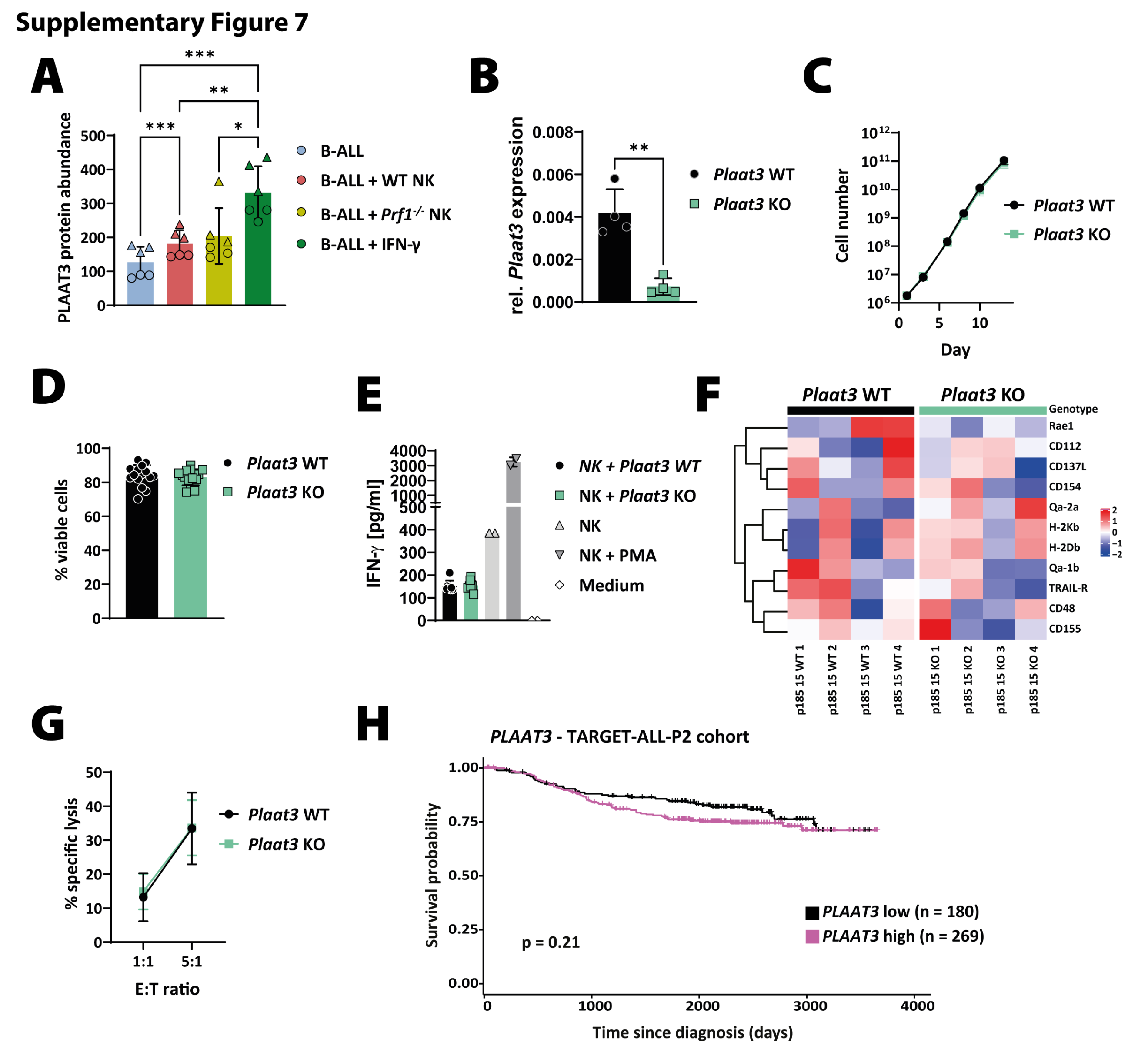


**Supplementary Figure S10: Generation of *Plaat3* KO B-ALL clones and their characterisation. (A)** Bar graph shows the protein abundance of PLAAT3 on day 14 of the same conditions as in the co-culture experiment described in Figure 4A with continuous IFN-γ treatment as additional condition. Two B-ALL cell lines (A in circles/D in triangles) were analysed by mass spectrometry in triplicates. Bars and error bars represent means of arbitrary units (AU) relative to the whole protein abundance ±SD; the significance was calculated by one-way ANOVA with Tukey´s multiple comparisons test. **(B)** *Plaat3* KO clones were generated by CRISPR/Cas9 genome editing in parental B-ALL cell lines. The gene modification was verified by TIDE sequencing (not shown) as well as by *Plaat3* mRNA expression analysed by qPCR. Bars and error bars represent means±SD; the significance was calculated using an unpaired t-test. **(C)** Growth curve of *Plaat3* WT and KO clones showing the proliferation in absolute cell numbers over 13 days. Shown are means±SD of n=5 clones per genotype in duplicates. **(D)** Cell viability of *Plaat3* WT and KO B-ALL clones. Shown are means±SD of n=5 clones per genotype of 3 independent experiments. **(E)** Measurement of IFN-γ secretion by mNK cells upon culturing in tumour cell conditioned medium for 48 h. As positive control mNK cells were activated with PMA/ionomycin. Bars and error bars represent mean±SD of n=4 clones per genotype measured in technical duplicates. **(F)** *Plaat3* WT and KO B-ALL cell lines (n=4 per genotype) were characterised by their surface expression of NK cell receptor ligands by flow cytometry. Columns represent different tumour cell clones ordered according to genotype and cell line. Data has been both scaled and clustered by row to highlight variations in marker expression across different samples. Red indicates higher and blue lower expression levels of surface ligands. **(G)** A 4 hour NK cytotoxicity assay was performed using WT and *Plaat3* KO B-ALL clones. Shown are means±SD of n=4 clones per genotype and 3 independent experiments. **(H)** Kaplan-Meier plot depicting survival probability of the TARGET-ALL-P2 patient cohort divided into *PLAAT3* high and low expressing groups (cut-off percentile = %). The graph was generated by using the online web tool: [cSurvival (ubc.ca)](https://tau.cmmt.ubc.ca/cSurvival/).
