# Supplementary material for: Natural killer cell–mediated cytotoxicity shapes the clonal evolution of B cell leukaemia
Source: Cancer Immunol Res. Author manuscript; Available in PMC 2025 Jan 14. (PMC7617306; doi:10.1158/2326-6066.CIR-24-0189)
Supplement: Supplementary Materials [file EMS201860-supplement-Supplementary_Materials.zip › supp_info_11.docx]

# Supplementary Figure S9


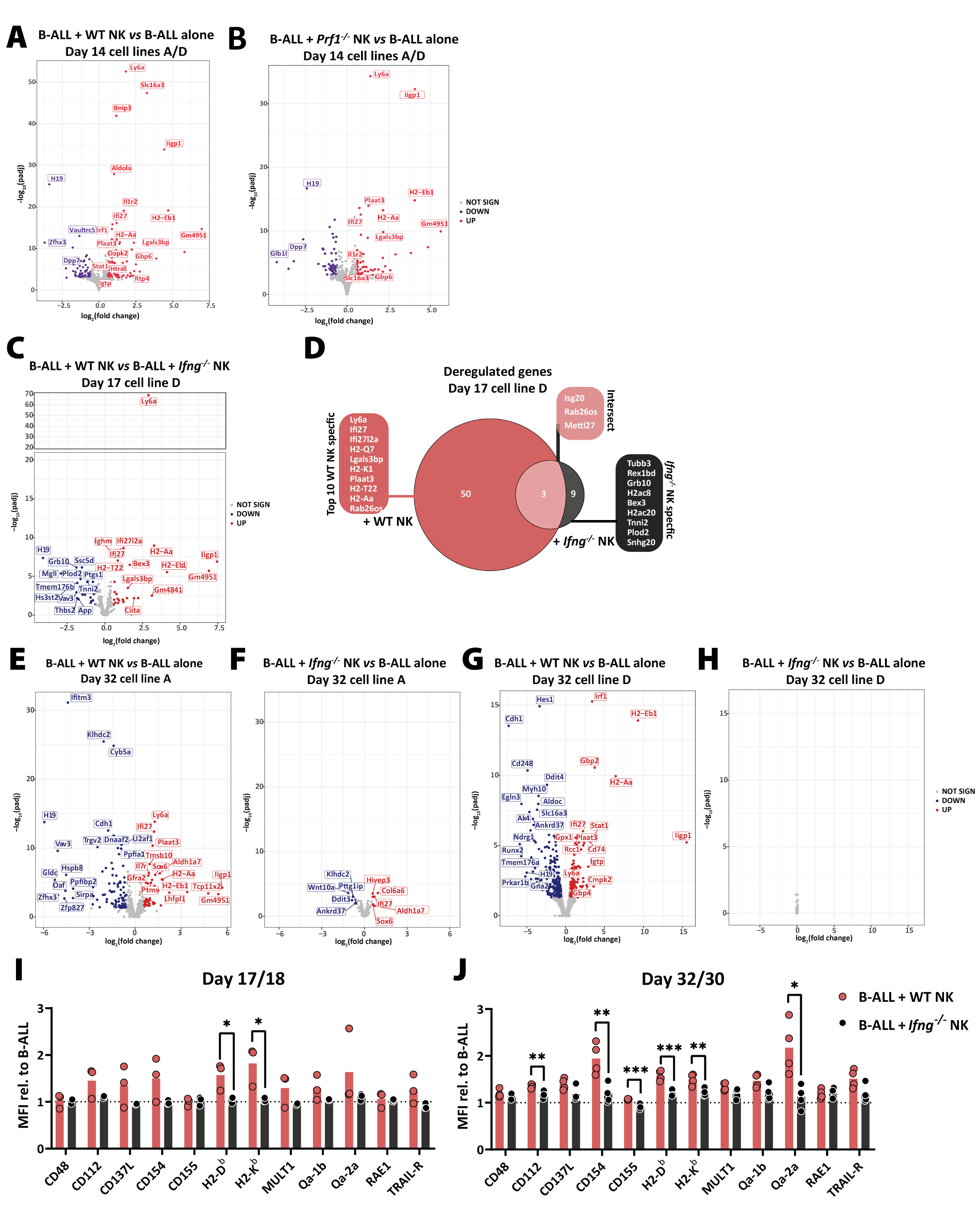


**Supplementary Figure S9: Profiles of tumour cells co-cultured with WT, *Prf1^-/-^* or *Ifng^-/-^* NK cells. (A&B)** The volcano plots depict DEGs in cell lines A/D on day 14 in **(A)** B-ALL + WT NK cells and **(B)** B-ALL + *Prf1^-/-^* NK cells compared to B-ALL alone. **(C)** The volcano plot shows the comparison of DEGs of B-ALL + WT NK *vs* B-ALL + *Ifng^-/-^* NK on day 17 in cell line D. **(D)** The Euler plot shows the comparison of DEGs in B-ALL + WT NK *vs* B-ALL alone with B-ALL + *Ifng^-/-^* NK *vs* B-ALL alone. Overlapping and WT or *Ifng^-/-^* NK cell specific genes are listed next to the Euler diagram. **(E-H)** The volcano plots depict DEGs in B-ALL cells upon 32 days of co-culture with WT or *Ifng^-/-^* NK cells in indicated cell lines. **(I&J)** The expression of inhibitory and activating NK cell receptor ligands on B-ALL cells co-cultured with WT or *Ifng^-/-^* NK cells was measured by flow cytometry on the indicated days. Shown are median fluorescence intensities (MFI) relative to B-ALL alone on the respective day. Shown are the means of n=3-4 measurements of cell line D from 2 individual experiments. Statistics were calculated by using an unpaired t-test.
