# Supplementary material for: Natural killer cell–mediated cytotoxicity shapes the clonal evolution of B cell leukaemia
Source: Cancer Immunol Res. Author manuscript; Available in PMC 2025 Jan 14. (PMC7617306; doi:10.1158/2326-6066.CIR-24-0189)
Supplement: Supplementary Materials [file EMS201860-supplement-Supplementary_Materials.zip › supp_info_10.docx]

# Supplementary Figure S8

**
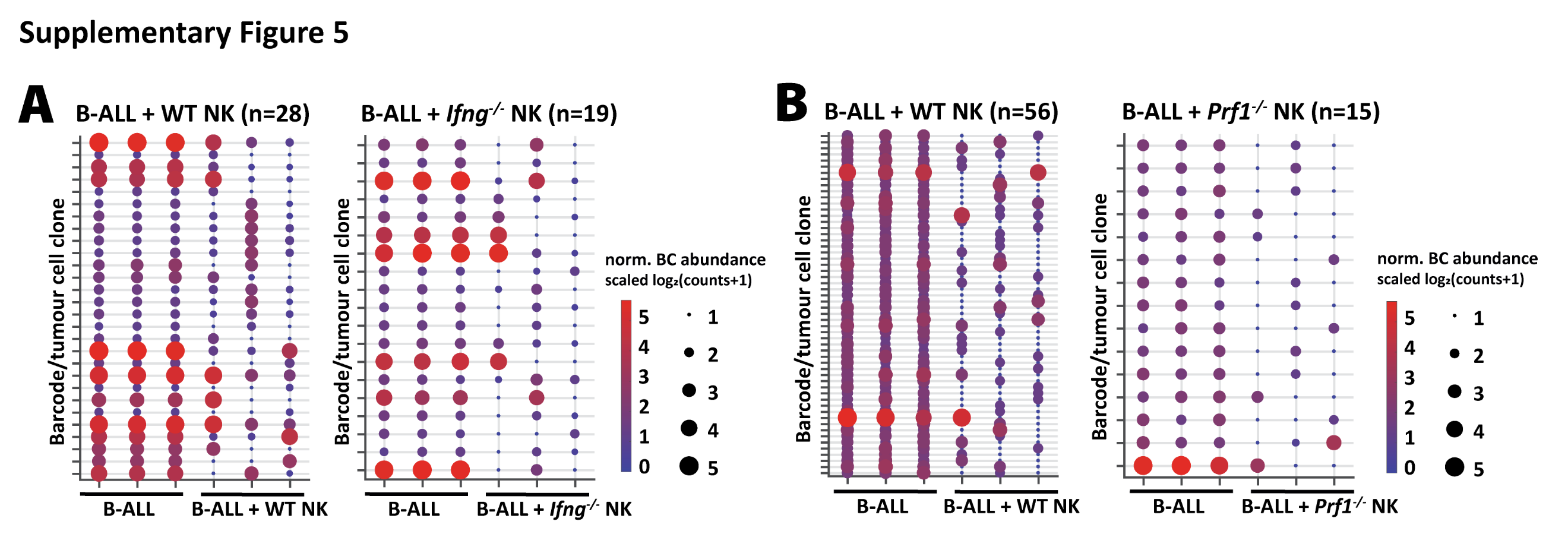
Supplementary Figure S8:** **Secondary resistant B-ALL cell clones.** The bubble plots show secondary resistant tumour cell clones determined in **(A)** B-ALL + WT or B-ALL + *Ifng^-/-^* NK cell conditions on day 17 and in **(B)** B-ALL + WT or B-ALL + *Prf1^-/-^* NK cell conditions on day 14. The x-axis shows the three individual wells of each condition, while each row on the y-axis shows an individual tumour cell clone. The corresponding abundance of these particular subclones in the B-ALL alone condition is shown on the left of each panel to highlight that the abundance of these subclones is equal in the control wells. Dot size and colour depicts normalised barcode abundance in cell line A.
