# Supplementary material for: Natural killer cell–mediated cytotoxicity shapes the clonal evolution of B cell leukaemia
Source: Cancer Immunol Res. Author manuscript; Available in PMC 2025 Jan 14. (PMC7617306; doi:10.1158/2326-6066.CIR-24-0189)
Supplement: Supplementary Materials [file EMS201860-supplement-Supplementary_Materials.zip › supp_info_8.docx]

# Supplementary Figure S6


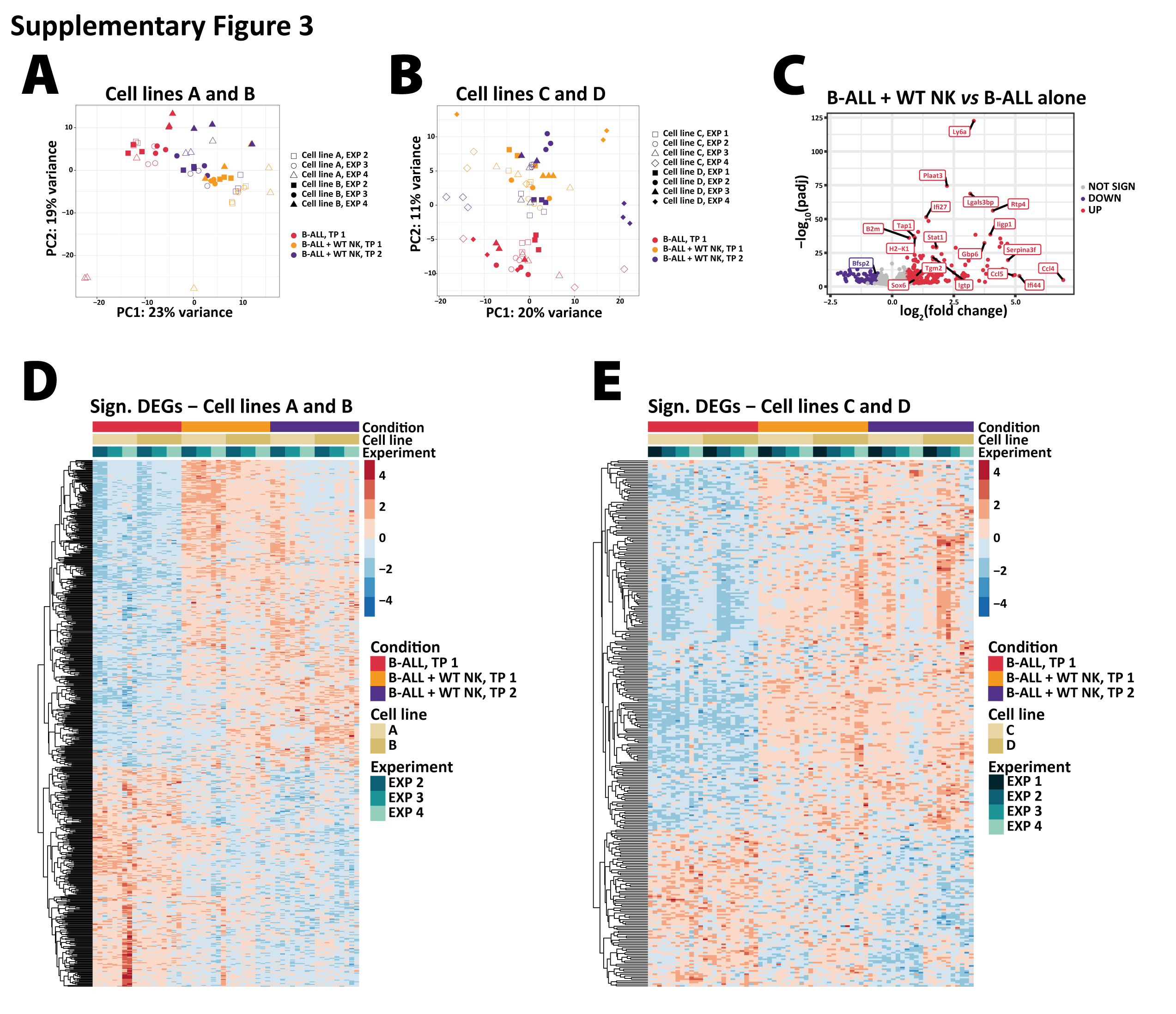


**Supplementary Figure S6:** **Analysis of differentially expressed genes in NK cell resistant tumour cells.** **(A&B)** The PCA plot illustrates the transcriptomic relatedness among cell lines **(A)** A/B and **(B)** C/D and conditions (B-ALL alone, B-ALL + WT NK cells on time point 1 (TP1) and TP2) in the long-term co-culture experiments, summarising 3 or 4 independent experiments, respectively. Data was batch corrected for experiments. **(C)** The volcano plot of the RNA sequencing analysis of cell lines C/D shows DEGs of B-ALL + WT NK co-cultured cells (TP2) versus B-ALL alone samples. **(D&E)** The heat maps summarise DEGs of cell lines **(D)** A/B (n=3 experiments) and **(E)** C/D (n=4 experiments). Heat maps were clustered by condition, cell line and experiment. Z-score log_2_(fold change) is shown and up- or downregulated genes are depicted in red and blue colour, respectively.
