# Supplementary material for: Natural killer cell–mediated cytotoxicity shapes the clonal evolution of B cell leukaemia
Source: Cancer Immunol Res. Author manuscript; Available in PMC 2025 Jan 14. (PMC7617306; doi:10.1158/2326-6066.CIR-24-0189)
Supplement: Supplementary Materials [file EMS201860-supplement-Supplementary_Materials.zip › supp_info_9.docx]

# Supplementary Figure S7**
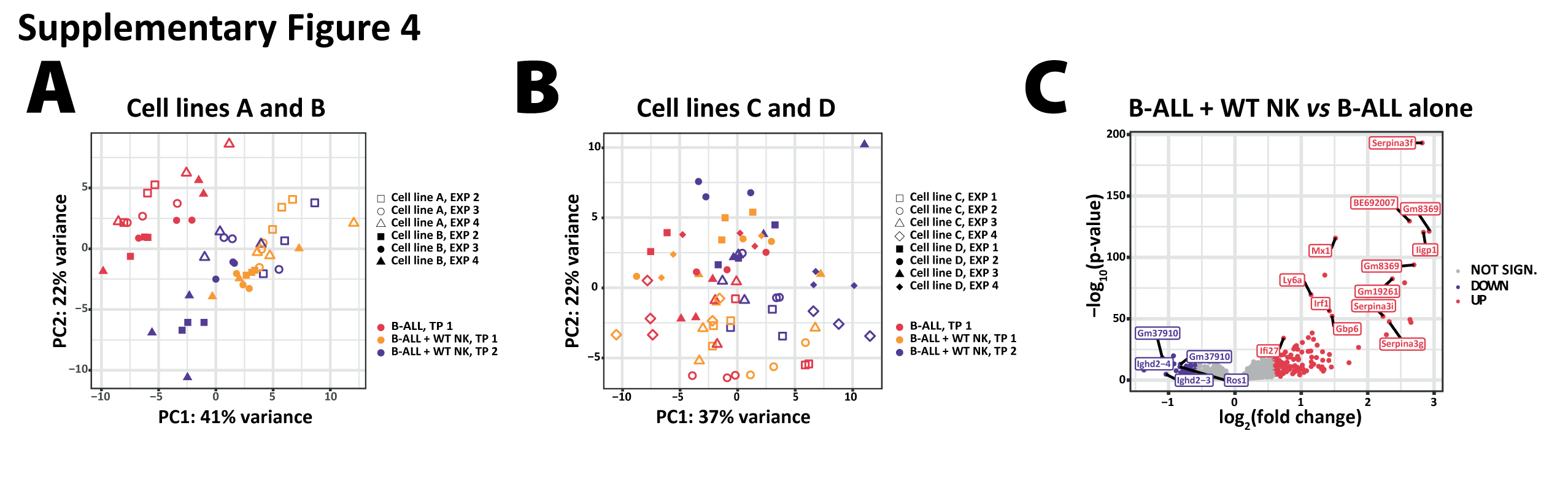
**

**Supplementary Figure S7:** **Analysis of differentially accessible genes in NK cell resistant tumour cells.** **(A&B)** PCA plots from the ATAC sequencing analysis compares the similarities of the chromatin accessibility of the cell lines and the different conditions. Cell lines A/B are shown in **(A)** and C/D in **(B)** summarising 3 and 4 independent experiments, respectively. **(C)** The DARs in B-ALL + WT NK cells versus B-ALL alone were compared and are shown in a volcano plot summarising 4 independent experiments using cell lines C/D.
