# Supplementary material for: Natural killer cell–mediated cytotoxicity shapes the clonal evolution of B cell leukaemia
Source: Cancer Immunol Res. Author manuscript; Available in PMC 2025 Jan 14. (PMC7617306; doi:10.1158/2326-6066.CIR-24-0189)
Supplement: Supplementary Materials [file EMS201860-supplement-Supplementary_Materials.zip › supp_info_7.docx]

# Supplementary Figure S5


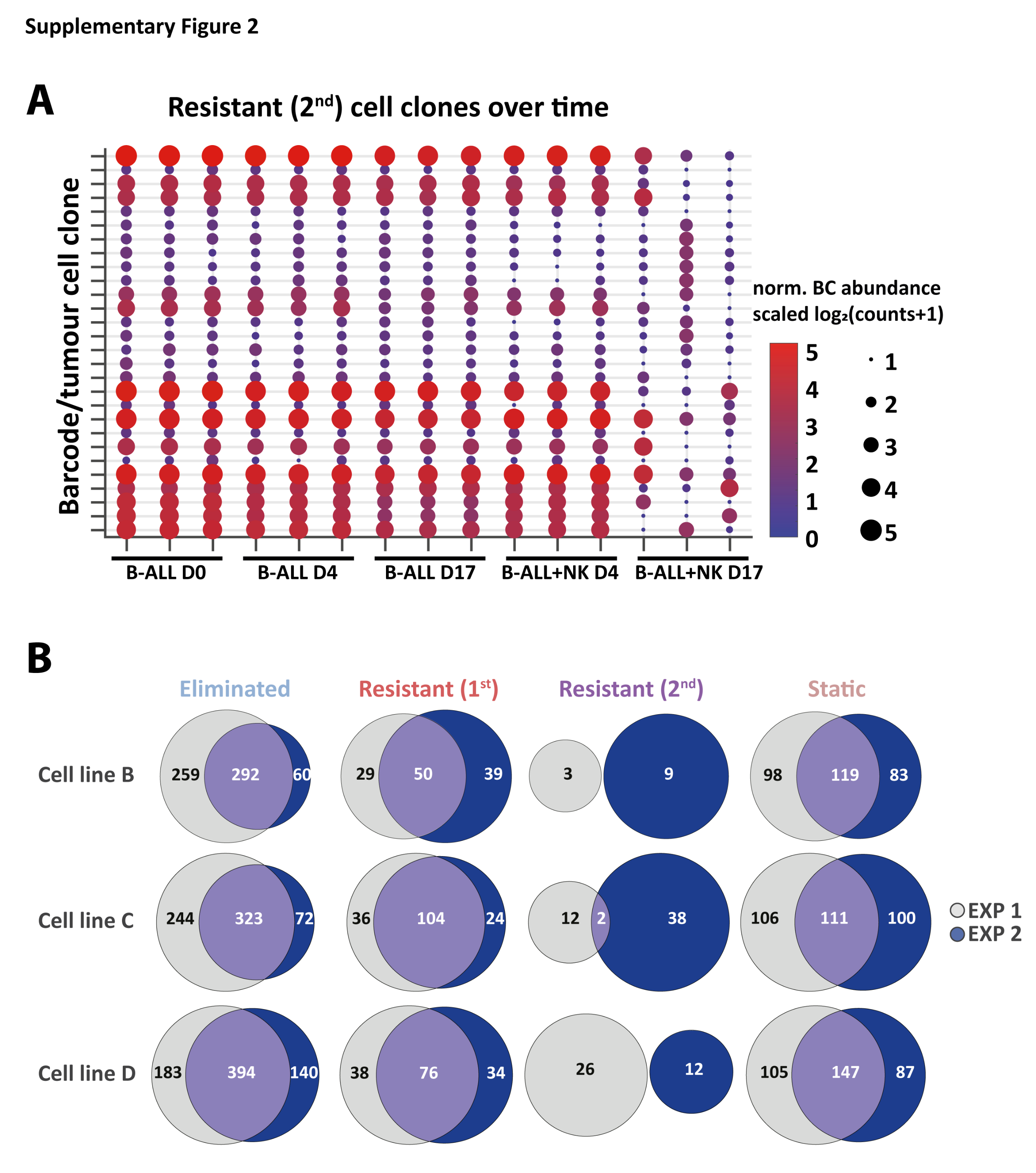


**Supplementary Figure S5: Extended quantification of NK cell-mediated cancer immunoediting *in vitro***. According to our hypothesis, the abundance of each barcoded tumour cell clone can be significantly higher (primary resistant) or lower (eliminated), unchanged (static) or show a high variability (secondary resistant) after extended NK cell co-culture. Each experiment was performed at least two times using the same barcoded parental cell line. **(A)** Bubble plot showing secondary resistant tumour cell clones on day 0, 4 and 17 related to data shown in Figure 4H. Depicted are B-ALL alone samples and B-ALL + NK cells in biological triplicates of cell line A. Dot size and colour show normalised barcode abundance. **(B)** The Euler diagrams compare two individual experiments of cell lines B, C and D (A is shown in Figure 2). The tumour cell clones that were categorised as eliminated, primary resistant, and static showed a relatively high overlap between the two independent experiments. In contrast, secondary resistant clones were less likely to stem from the same original clone when comparing independent experiments.
