# Supplementary material for: Natural killer cell–mediated cytotoxicity shapes the clonal evolution of B cell leukaemia
Source: Cancer Immunol Res. Author manuscript; Available in PMC 2025 Jan 14. (PMC7617306; doi:10.1158/2326-6066.CIR-24-0189)
Supplement: Supplementary Materials [file EMS201860-supplement-Supplementary_Materials.zip › supp_info_5.docx]

# Supplementary Figure S3


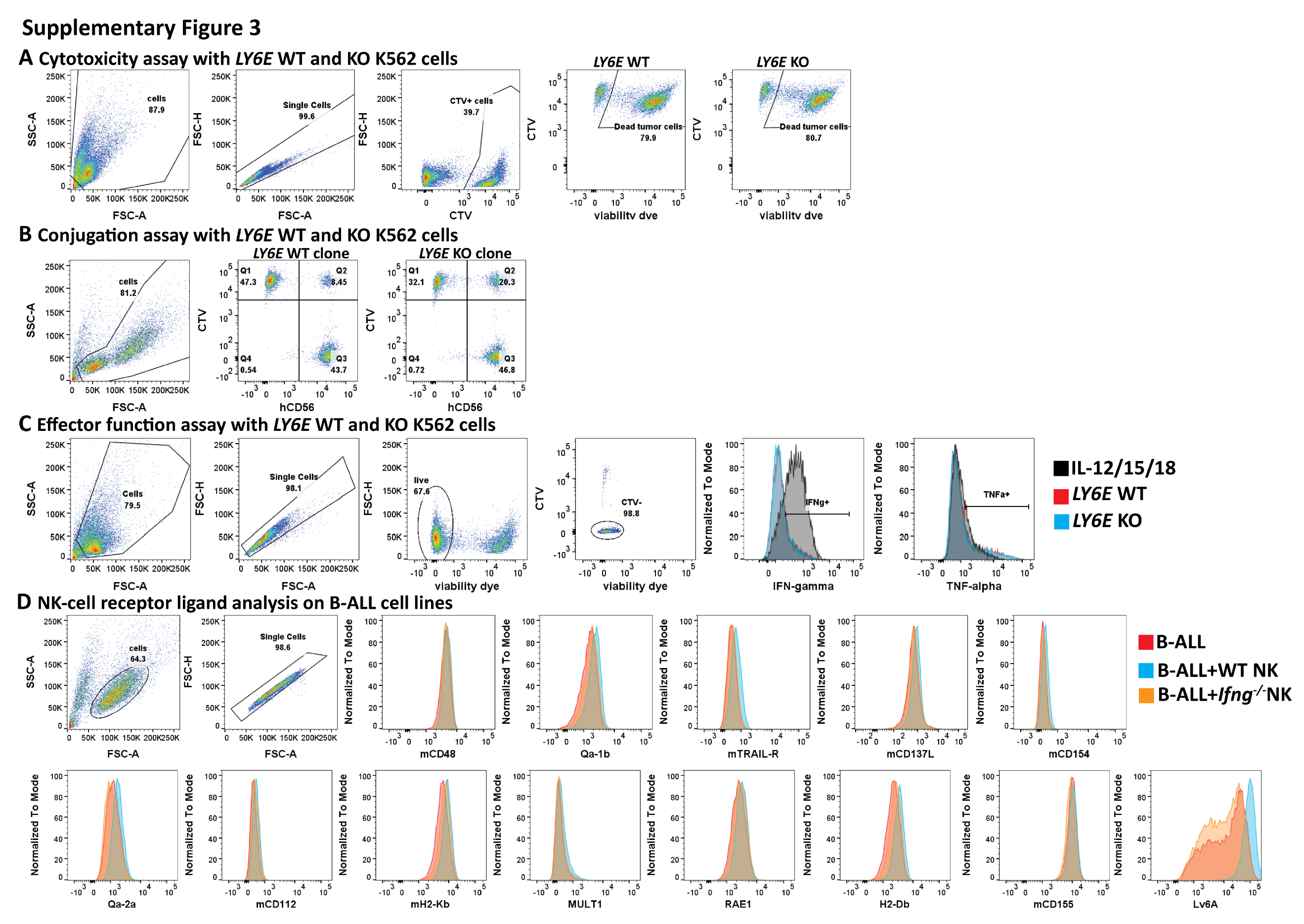


**Supplementary Figure S3: Flow cytometric analyses of human data sets and the NK-cell receptor ligand analyses on mouse B-ALL cell lines. (A)** This panel depicts a representative gating strategy for the cytotoxicity assays investigating the NK-cell susceptibility of *LY6E* WT and *LY6E* KO K562 cell clones shown in Figure 6G. The cell debris were excluded in the FSC-A and SSC-A dot plot, single cells were selected in the FSC-A and FSC-H dot plot and further, CTV^+^ tumour cells were separated from the CTV^-^ NK cells. The NK cell killing was quantified by gating on the dead viability dye^+^ CTV^+^ tumour cells. The fourth and fifth dot plot from the left depict a representative example of the NK cell-mediated killing of *LY6E* WT and *LY6E* KO cells at an E:T ratio of 1:1. **(B)** This panel depicts the gating strategy of the conjugation formation analysis shown in Figure 6H. Cell debris were excluded in the FSC-A and SSC-A dot plot and all cells, also doublets, were included in further analysis. Conjugates were detected by their double positivity of CTV (tumour cells) and hCD56 (NK cells). The second and third dot plot from the left show a representative picture of the conjugates formed by NK cells with *LY6E* WT and *LY6E* KO cells, respectively. **(C)** This panel shows the gating strategy of the NK-cell effector function assay in Supplementary Figure S11. After doublet exclusion, living cells were detected by their viability dye negativity. Further, CTV^+^ tumour cells were excluded, and the CTV^-^ NK cells were analysed for their IFN-γ and TNF-α production by gating on the IFN-γ^+^ and TNF-α^+^ NK cells. Representative histograms show the production of IFN-γ or TNF-α in NK cells co-cultured with *LY6E* WT K562 cells (red), LY6E *KO* K562 cells (blue) and after stimulation with IL-12, IL-15 and IL-18 (black). **(D)** Flow cytometric analysis of NK-cell receptor ligand expression on B-ALL cell lines shown in Figure 1B, 5B and 5I, Supplementary Figure S4A, S9I, S9J and S10F. Single living cells were gated according to size and granularity in FSC-A, SSC-A and FSC-H plots. The cell surface marker expression was quantified by MFI. The histograms show all the analysed surface markers separately and depict a representative example of the data shown in Figure 5B and Supplementary Figure S9J, whereby B-ALL cells cultured alone are indicated in red, B-ALL+WT NK cells in blue and B-ALL+*Ifng^-/-^* NK cells in orange.
