# Supplementary material for: Natural killer cell–mediated cytotoxicity shapes the clonal evolution of B cell leukaemia
Source: Cancer Immunol Res. Author manuscript; Available in PMC 2025 Jan 14. (PMC7617306; doi:10.1158/2326-6066.CIR-24-0189)
Supplement: Supplementary Materials [file EMS201860-supplement-Supplementary_Materials.zip › supp_info_6.docx]

# Supplementary Figure S4


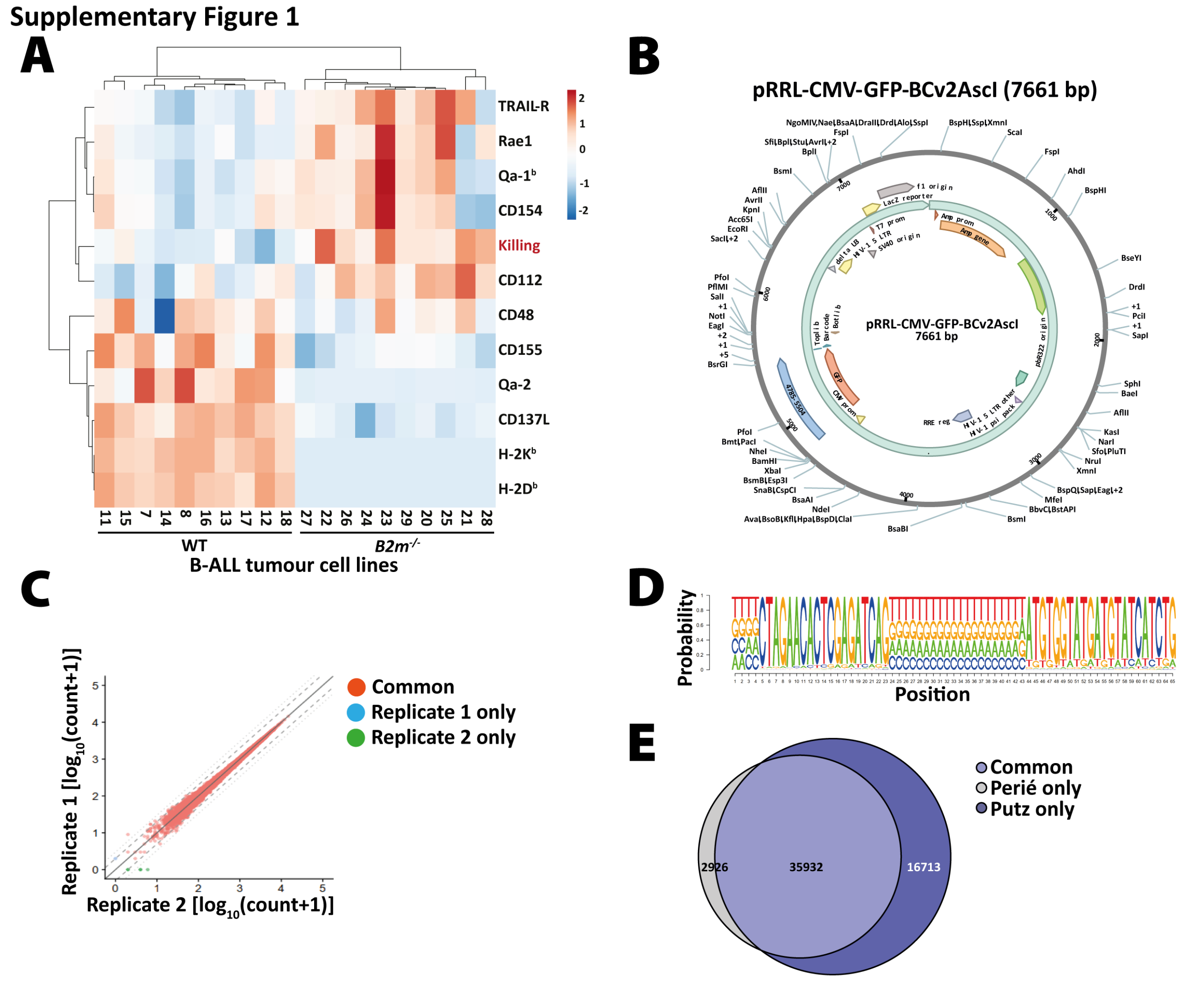


**Supplementary Figure S4:** **B-ALL cell line characterisation and details about the barcode library.** **(A)** WT (see **Figure 1B**) and *B2m^-^****^/-^*** B-ALL cell lines were characterised regarding the surface expression of NK cell receptor ligands and their susceptibility towards NK cell killing (depicted here is the cytotoxicity at E:T=10:1). Rows were centred; unit variance scaling was applied to rows. Both rows and columns were clustered using correlation distance and average linkage. **(B)** Plasmid vector map of the pRRL-CMV-*GFP*-BCv2Ascl barcode library plasmid. In the inner circle the CMV promoter, the *GFP* gene, barcode nucleotides (n=21) with Toplib and Botlib primers and the ampicillin resistance are highlighted. Restriction enzyme sites are depicted on the outer circle. **(C)** The barcode library was sequenced in technical replicates after plasmid transformation, MaxiPrep and following a 3-step nested PCR library preparation protocol. After raw data processing and filtration for valid barcodes, the barcodes of the biological replicates were compared and overlapped well. Common barcodes are shown in orange; non-overlapping barcodes from replicates 1 or 2 are show in blue or green, respectively. **(D)** The graph shows a representative sequence of one replicate of the barcode library (n=4). Positions 1-4 represent the random nucleotide insertion after using the BC1v2DS_For primer in PCR#2. Positions 5-23 and 45-65 are the constant sequences before and after the barcode, respectively. The 21 nucleotides from positions 24-44 represent the barcode sequence. **(E)** The barcode reference library generated in-house (Putz laboratory) was compared to the one used in the Perié laboratory. Both libraries, which were sequenced independently, were overlapping to a high degree. The Putz library detected more barcodes, which was likely due to the higher sequencing depth.
