# Supplementary material for: Natural killer cell–mediated cytotoxicity shapes the clonal evolution of B cell leukaemia
Source: Cancer Immunol Res. Author manuscript; Available in PMC 2025 Jan 14. (PMC7617306; doi:10.1158/2326-6066.CIR-24-0189)
Supplement: Supplementary Materials [file EMS201860-supplement-Supplementary_Materials.zip › supp_info_4.docx]

# Supplementary Figure S2


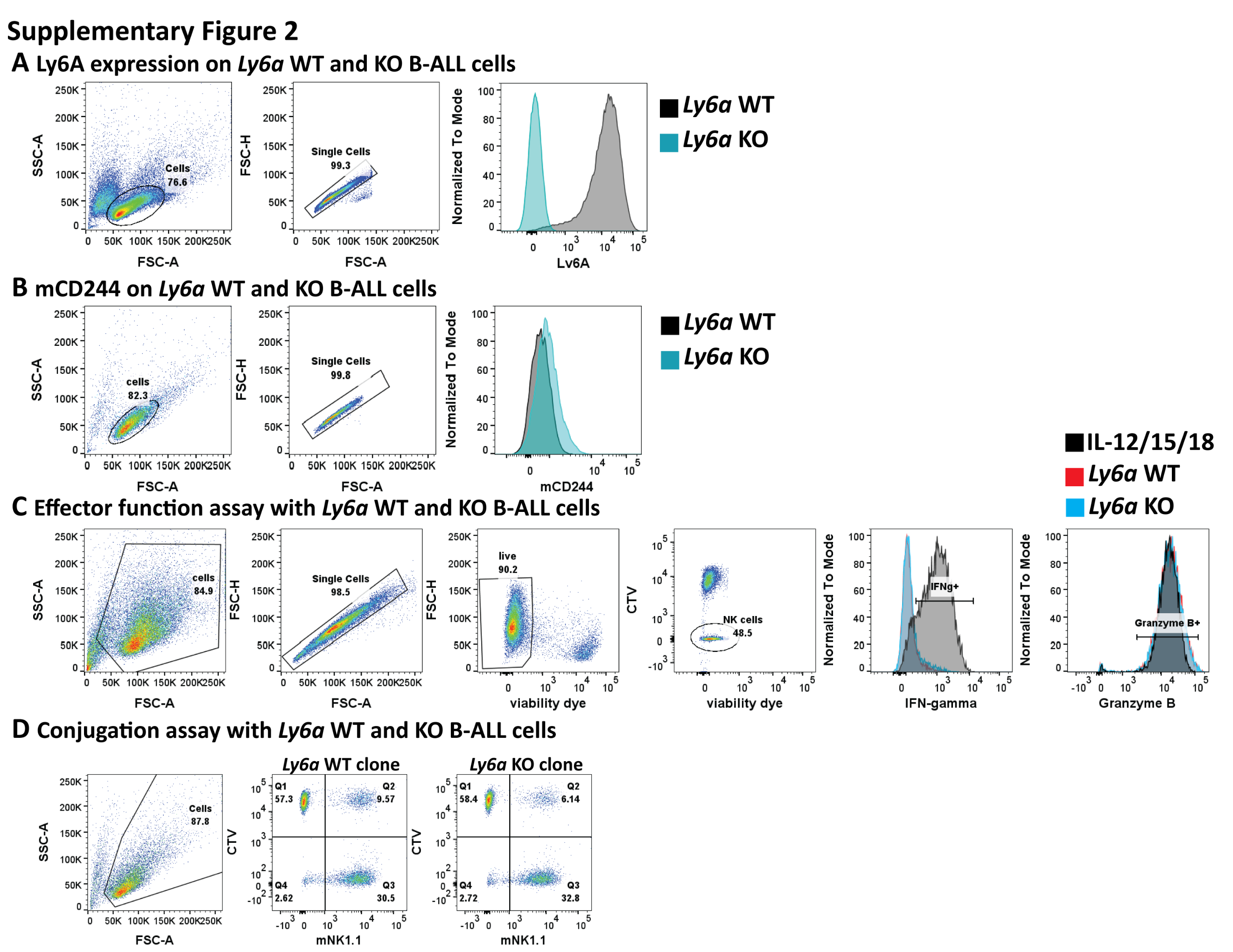


**Supplementary Figure S2: Flow cytometric analysis and gating strategies shown in Figure 5.** **(A-C)** Single living cells were gated according to size and granularity in FSC-A, SSC-A and FSC-H plots. **(A)** The histogram depicts a representative example of the Ly6A expression of a *Ly6a* WT (black) and *Ly6a* KO (green) clone shown in Figure 5C. **(B)** The histogram shows the expression of CD244 on a representative *Ly6a* WT (black) and *Ly6a* KO (green) clone shown in Figure 5J. **(C)** Dot plots depict the gating strategy of the NK-cell effector function assay shown in Figure 5K & 5L. After doublet exclusion living cells were detected by their viability dye negativity. CTV^+^ tumour cells were excluded, and the CTV^-^ NK cells were analysed for their IFN-γ and granzyme B production by gating on the IFN-γ^+^ and granzyme B^+^ NK cells. Representative histograms show IFN-γ or granzyme B expression in NK cells co-incubated with *Ly6a* WT cells (red), Ly6a *KO* cells (blue) of after stimulation with IL-12, IL-15 and IL-18 (black). The expression of granzyme B was quantified by the MFI of granzyme B^+^ NK cells. **(D)** Dot plots depict the gating of the conjugation formation analysis depicted in Figure 5M. Cell debris were excluded in the FSC-A and SSC-A dot plot and all cells, also doublets, were included in further analysis. Conjugates were detected by their double positivity of CTV (tumour cells) and mNK1.1 (NK cells). The second and third dot plot from the left show a representative picture of the conjugates formed by NK cells with *Ly6a* WT and *Ly6a* KO cells, respectively.
