# Supplementary material for: Natural killer cell–mediated cytotoxicity shapes the clonal evolution of B cell leukaemia
Source: Cancer Immunol Res. Author manuscript; Available in PMC 2025 Jan 14. (PMC7617306; doi:10.1158/2326-6066.CIR-24-0189)
Supplement: Supplementary Materials [file EMS201860-supplement-Supplementary_Materials.zip › supp_info_3.docx]

# Supplementary Figure S1

**
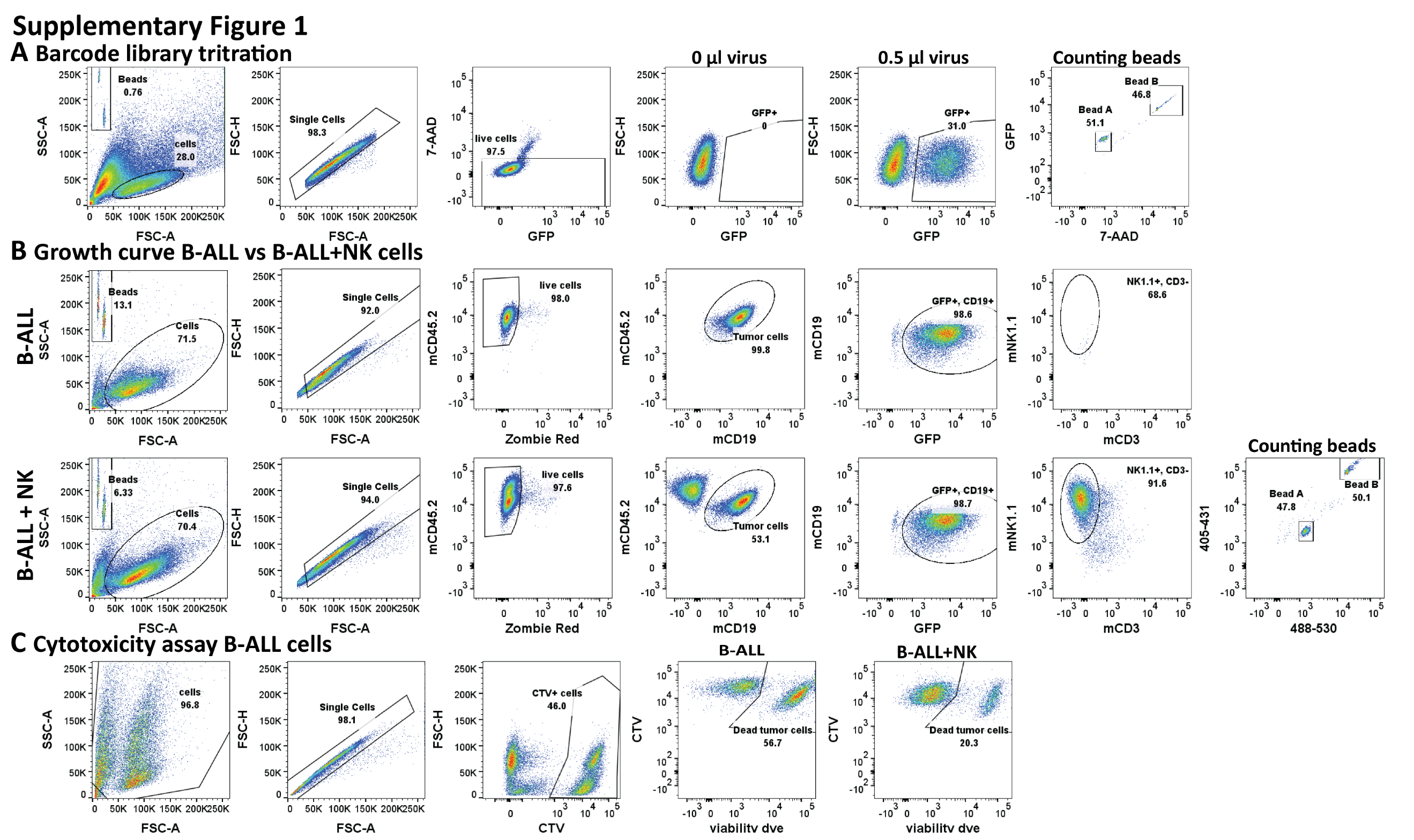
**

**Supplementary Figure S1:** **Flow cytometric analysis and gating strategies.** **(A&B)** Single living cells were gated according to size and granularity in FSC-A, SSC-A and FSC-H plots. **(A)** Dot plots depict the gating strategy for the barcode library titration in Figure 1C. After doublet exclusion, living cells were selected by their 7-AAD negativity. Further, the transduction rate was assessed by the percentage of GFP^+^ cells (exemplified by the addition of 0 µl and 0.5 µl virus). The cell number was quantified by using AccuCheck Counting Beads depicted in the right dot plot. **(B)** Shown is the gating strategy for the *in vitro* NK cell and tumour cell co-culture depicted in Figure 1E, 4A and 4C. After doublet exclusion, live cells were determined by their Zombie Red negativity. Further, the tumour cells and NK cells were gated as mCD19^+^ and mCD19^-^ cells, respectively. mCD19^+^GFP^+^ cells were determined as B-ALL cells. mCD19^-^ cells were further verified to express mNK1.1 and to lack mCD3. The upper panel depicts a representative gating strategy for the B-ALL alone condition and the lower panel for the B-ALL+NK cell condition. The cell number was quantified by using AccuCheck Counting Beads. **(C)** This panel depicts a representative gating strategy for the cytotoxicity assays with the B-ALL tumour cell lines shown in Figure 1F, 4B, 4D and 5N. The cell debris were excluded in the FSC-A and SSC-A dot plot, single cells were selected in the FSC-A and FSC-H dot plot and CTV^+^ tumour cells were separated from the CTV^-^ NK cells. The NK cell killing was quantified by gating on the dead viability dye^+^ CTV^+^ tumour cells. The fourth and fifth dot plot from the left depict a representative example of the data shown in Figure 1F (day 29; E:T ratio = 5:1) comparing the NK cell-mediated killing of B-ALL cells cultured for 20 days in the presence or absence of NK cells.
