# Supplementary material for: Natural killer cell–mediated cytotoxicity shapes the clonal evolution of B cell leukaemia
Source: Cancer Immunol Res. Author manuscript; Available in PMC 2025 Jan 14. (PMC7617306; doi:10.1158/2326-6066.CIR-24-0189)
Supplement: Supplementary Materials [file EMS201860-supplement-Supplementary_Materials.zip › supp_info_13.docx]

# Supplementary Figure S11


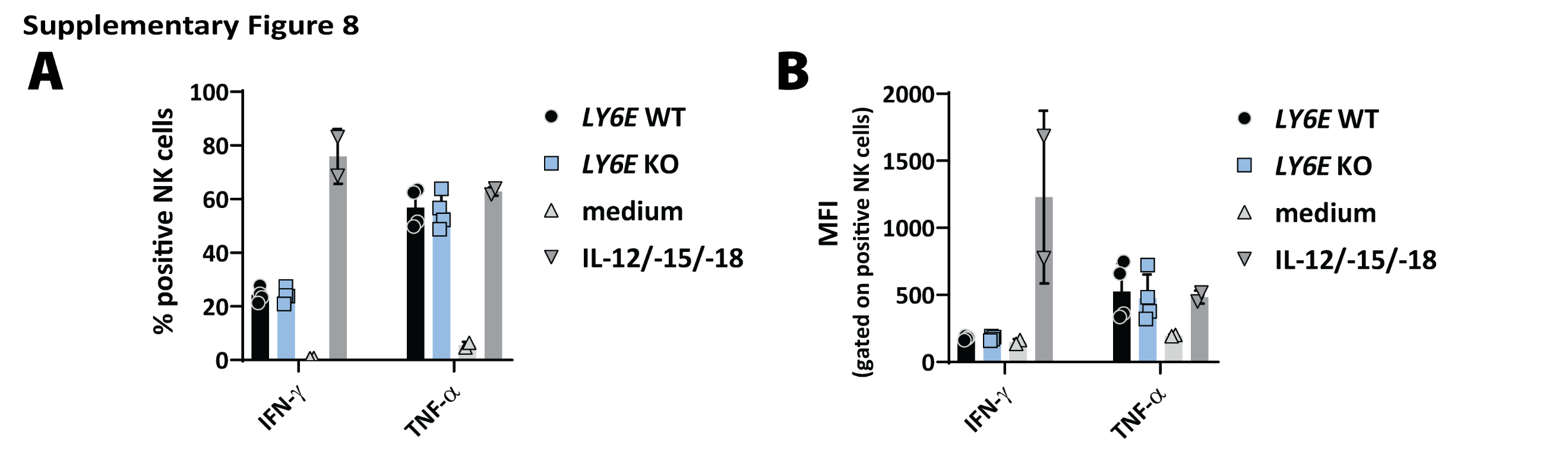


**Supplementary Figure S11: The expression of LY6E on K562 does not impact on IFN-γ or TNF-α production in NK cells**. IFN-γ and TNF-α production was measured in NK cells 4 hours after co-culture with *LY6E* WT or KO clones. Panel **(A)** shows the percentage, and **(B)** shows the MFI of cytokine^+^ NK cells. Bars and error bars represent mean±SD of n=2 clones per genotype measured in 2 individual experiments.
